# Identification of Novel Biomarkers for Evaluating Disease Severity in House-Dust-Mite-Induced Allergic Rhinitis by Serum Metabolomics

**DOI:** 10.1155/2021/5558458

**Published:** 2021-05-19

**Authors:** Shaobing Xie, Hua Zhang, Zhihai Xie, Yongzhen Liu, Kelei Gao, Junyi Zhang, Shumin Xie, Fengjun Wang, Ruohao Fan, Weihong Jiang

**Affiliations:** ^1^Department of Otolaryngology Head and Neck Surgery, Xiangya Hospital of Central South University, Changsha, Hunan, China; ^2^Hunan Province Key Laboratory of Otolaryngology Critical Diseases, Changsha, Hunan, China

## Abstract

The aim of this study was to identify differences in serum metabolomics profiles of house-dust-mite (HDM)-induced allergic rhinitis (AR) patients compared to controls and to explore novel biomarkers reflecting disease severity. Serum samples were collected from 29 healthy controls and HDM-induced 72 AR patients, including 30 mild patients (MAR) and 42 moderate to severe AR patients (MSAR). Metabolomics detection was performed, and orthogonal partial least square discriminate analysis was applied to assess the differences between AR patients and controls and for subgroups based on disease severity. These analysis results successfully revealed distinct metabolite signatures which distinguished MAR patients and MSAR patients from controls. MSAR patients also could be discriminated from MAR patients based on their metabolic fingerprints. Most observed metabolite changes were related to glycine, serine, and threonine metabolism, pyrimidine metabolism, sphingolipid metabolism, arginine and proline metabolism, and fatty acid metabolism. Levels of sarcosine, sphingosine-1-phosphate, cytidine, and linoleic acid significantly correlated with the total nasal symptom score and visual analogue scale in AR patients. These results suggest that metabolomics profiling may provide novel insights into the pathophysiological mechanisms of HDM-induced AR and contribute to its evaluation of disease severity.

## 1. Introduction

Allergic rhinitis (AR) is an IgE-mediated immunologic disease characterized by mucus hypersecretion and airway hyperresponsiveness caused by common allergens such as house dust mite (HDM), pollen, and animal dander [1, 2]. Among these allergies, HDM is the most common one, and HDM-induced AR is particularly troublesome, given the almost ubiquitous presence of HDMs in indoor environments worldwide [3]. Epidemiological studies showed that AR affected at least half a billion people worldwide, and more than half of them were moderate to severe [3]. In China, the prevalence of AR even rises to 34.3% of the general population, and rates still continue to increase [4, 5]. Although not life threatening, AR exhibits a negative influence on people's quality of life and their work production and brings about a high medical cost on individuals and society. Currently, AR is subdivided into intermittent AR and persistent AR according to the allergic rhinitis and its impact on asthma (ARIA) classification [6], and persistent AR is further grouped into mild AR (MAR) and moderate to severe AR (MSAR) based on the severity scale. The immunological underpinnings and their associations with disease severity have been a hot topic of significant research, but remain poorly clarified.

Previous publications reported that AR was a heterogeneous disease with a wide degree of severity; there was no available objective indicator or biological marker that is specific for its disease activity [7, 8]. Current monitoring of the severity of this clinical disorder relies primarily on the subjective clinical symptom score, which is relatively insensitive, particularly in children [9, 10]. For most AR patients, they often do not recognize how severe their symptoms are because of progressive tolerance to these symptoms. Furthermore, self-reported symptoms used by physicians to guide treatment and evaluate the therapeutic effect are likely imprecise. Potentially, this also can lead to growing costs of healthcare and wasted resources [7]. In addition, clinical research in AR is hampered because of a lack of sensitive biological measures of disease severity. Therefore, identification of biomarkers of disease severity is urgently required to improve patient management and then accelerate drug development in AR.

Metabolomics, a branch of omics science that systematically analyzes the concentration profiles of low molecular weight endogenous metabolites generated by living systems, is a promising approach to identify new biomarkers and novel metabolic pathways for several diseases, simultaneously providing new insights into the underlying pathophysiological mechanisms [11, 12]. Recently, several studies have employed metabolomics technologies to explore the metabolic changes in asthma, pneumonia, and chronic obstructive pulmonary disease and successfully identified some potential biomarkers and key metabolic pathways [13–15]. However, no previous study has focused on metabolite and metabolic pathway changes in the serum of AR patients, especially regarding the disease severity.

Therefore, the primary aim of this study was to explore the metabolic profiling of HDM-induced AR patients and determine the relationship between the metabolite changes and clinical severity, thus to provide new insights into the complex pathophysiological mechanisms and monitor disease activity. In this study, ultrahigh-performance liquid chromatography mass spectrometry (UHPLC-MS) was performed to detect metabolites in serum samples of MSAR, in comparison with MAR and healthy controls. In addition, linear regression analysis was performed to evaluate the correlation between metabolites and disease severity.

## 2. Materials and Methods

### 2.1. Participants and Settings

This is a prospective study with 101 participants recruited between June 2018 and January 2019. All participants were divided into three groups: the MAR group (*n* = 30), MSAR group (*n* = 42), and control group (*n* = 29). The diagnosis of HDM-induced AR was done on the basis of medical history and allergic symptoms (sneezing, rhinorrhea, nasal congestion, and nasal itching) for at least 2 years, positive skin test results (a mean wheal diameter > 3 mm), and positive specific IgE to HDM (>0.35 IU/mL). Patients with persistent AR were categorized into MAR and MSAR according to ARIA criteria [6]. Exclusion criteria included current smoking, other allergic diseases, systemic steroid treatment, inflammatory or septic diseases, autoimmune diseases, cardiovascular diseases and liver and kidney dysfunction, age < 18 years, pregnant condition, immunotherapy, and use of antiallergic drugs during the 1 month that preceded the study. The total IgE levels, specific IgE to HDM levels, blood eosinophil count, and demographic information of the study subjects were collected, including gender, age, body mass index (BMI), and the duration of disease duration. All participants scored their symptoms by using the widely accepted total nasal symptom score (TNSS) and visual analogue scale (VAS) which were described by previous studies [7, 16]. The TNSS is the sum of 4 individual symptom scores for sneezing, rhinorrhea, nasal congestion, and nasal itching, and each symptom score was regarded on a 4-point scale from 0 to 3 (0 = no symptoms; 1 = minimal, well-tolerated symptoms; 2 = bothersome but tolerated symptoms; and 3 = severe and hard to tolerate symptoms). In addition, the global disease severity over the last week was evaluated by a VAS (0-10 cm: where 0 is no symptoms and 10 cm is the maximum severity). The detailed clinical information of the recruited participants among three groups is described in Table 1.

### 2.2. Serum Sample Collection and Preparation

Serum samples were collected from HDM-induced AR patients and healthy controls with serum separator tubes without anticoagulation or coagulant before breakfast, and serum samples were stored for 1 hour at room temperature. All blood samples were centrifuged at 4°C (3000 rpm for 10 minutes); then, we collected the supernatants and stored them at -80°C in equal aliquots for subsequent detection and analysis. The serum samples were prepared for UHPLC-MS analysis by mixing 100 *μ*L of serum sample with 300 *μ*L methanol containing an internal standard (L-2-chlorophenylalanine, 2 *μ*g/mL). After a 30-second vortex, the samples were sonicated for 10 minutes in ice-water bath. Then, the samples were incubated at -40°C for 1 hour and centrifuged at 12000 rpm for 15 minutes at 4°C. 100 *μ*L of the supernatant was transferred to a fresh glass vial for UHPLC-MS analysis [16]. The quality control (QC) sample was prepared by mixing an equal aliquot of the supernatants from all the samples and used to evaluate the reproducibility and reliability of the UHPLC-MS analytical system as described by the previous study [17].

### 2.3. Untargeted UHPLC-MS Metabolomics

Samples were analyzed on a 1290 Infinity series UHPLC System (Waters Corporation, Milford, MA, USA) as previous study described [17]. Briefly, 10 *μ*L of reconstituted sample was injected on a UPLC BEH Amide column (2.1 mm × 100 mm, 1.7 *μ*m). The mobile phase consisted of 25 mmol/L ammonium acetate and 25 mmol/L ammonia hydroxide in water (pH = 9.75) (A) and acetonitrile (B). Each sample was analyzed in the positive ion mode and negative ion mode. The Triple TOF 6600 mass spectrometry (AB Sciex, Boston, MA, USA) was used for its ability to acquire MS/MS spectra on an information-dependent basis (IDA) during an LC/MS experiment. In this mode, the acquisition software (Analyst TF 1.7, AB Sciex, Framingham, MA, USA) continuously evaluates the full scan survey MS data as it collects and triggers the acquisition of MS/MS spectra depending on the preselected criteria. In each cycle, the most intensive 12 precursor ions with intensity above 100 were chosen for MS/MS at a collision energy (CE) of 30 eV. The cycle time was 0.56 second. Electrospray ionization (ESI) source conditions were set as follows: gas 1 as 60 psi, gas 2 as 60 psi, curtain gas as 35 psi, source temperature as 600°C, declustering potential as 60 V, and ion spray voltage floating (ISVF) as 5000 V or -4000 V in the positive or negative modes, respectively.

### 2.4. Data Processing and Analysis

MS raw data (.wiff) files were converted to the mzXML format by Proteo Wizard and processed by R package XCMS V3.2. The process includes peak deconvolution, alignment, and integration. Minfrac and cut-off are set as 0.5 and 0.3, respectively, as before [18]. In-house MS2 database was applied for metabolite identification [19]. The resultant data was exported to SIMCA (version 14.1, Umetrics, Umea, Sweden) for multivariate analysis. Orthogonal partial least square discriminant analysis (OPLS-DA) was performed to find potential biomarkers that contributed to the metabolic difference between the groups [20]. The quality of the models was validated by *R*^2^*Y*(cum) (goodness of fit) and *Q*2(cum) (goodness of prediction). Meanwhile, the 7-fold cross-validation and 200 permutation tests were conducted to reduce the risk of overfitting and the possibilities of false-positive findings. Metabolites contributing were selected according to the variable importance for project (VIP) values (VIP > 1.0) and *P* values (*P* < 0.05) [21]. To determine the performance of the identified combination, receiver operating characteristics (ROC) analysis was conducted, and area under the curve (AUC) was calculated to assess the sensitivity and specificity. In order to gain insight into the underlying metabolic mechanisms associated with AR and its severity, the metabolic pathway was analyzed in both ion modes using MetaboAnalyst 3.0.

### 2.5. Statistical Analysis

Normally distributed variables were displayed as mean ± standard deviation (SD), and one-way analysis of variance (ANOVA) was performed for comparison among three groups, Student's *t* test was utilized for comparison between two groups; nonnormally distributed data were described as median and interquartile range, and Kruskal–Wallis *H* test and Mann–Whitney *U* test were utilized for comparison among three groups and between two groups, respectively. Categorical variables are described as number (%) and compared utilizing Chi-square test. To explore the correlation between the levels of metabolites and the severity of AR, Spearman's correlation analysis was conducted. Differences were considered as significant when *P* < 0.05. All the above statistical analyses were carried out using SPSS statistics software version19.0 (IBM, Chicago, IL, USA).

## 3. Results

### 3.1. Baseline Characteristics of All Participants

The main characteristics and clinical information of the participants are shown in Table 1. No statistically significant difference was observed in gender, age, BMI, and disease duration among the three groups. In comparison with the control group and MAR group, the MSAR group showed higher levels of serum total IgE, specific IgE to HDM, blood eosinophil counts, TNSS, and VAS (all *P* < 0.001).

### 3.2. Metabolomics Profiling of MAR vs. Health Controls

**Figure 1:**
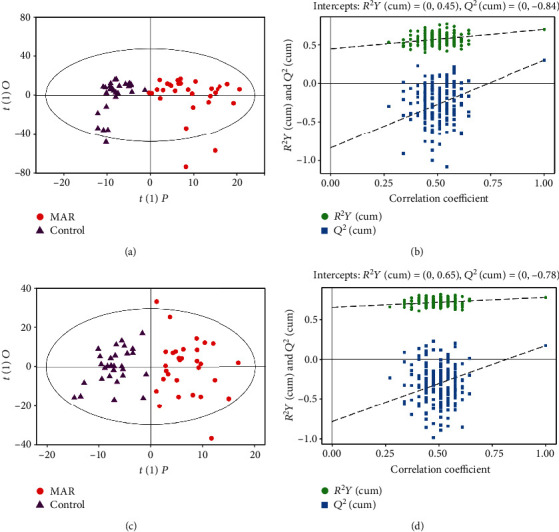
Metabolomics analysis of serum samples from the MAR group and control group. Score plot with OPLS-DA in the ESI+ (a) and ESI- (c) modes in MAR patients and controls. Permutation test of the OPLS-DA model in the ESI+ (b) and ESI- (d) modes. The values of *R*^2^*Y* and *Q*^2^ represent the goodness of fit and predictability of the model, respectively. OPLS-DA: orthogonal partial least square discriminant analysis; ESI: electrospray ionization; MAR: mild allergic rhinitis.

**Figure 2:**
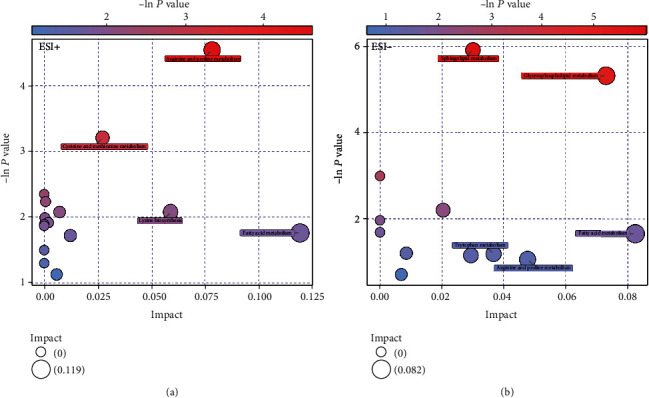
Metabolic function bubble chart based on the serum metabolomics profiles between MAR patients and controls in ESI+ (a) and ESI- (b) modes. ESI: electrospray ionization; MAR: mild allergic rhinitis.

OPLS-DA models showed a clear and distinctive clustering between the MAR group and control group in both the ESI+ and ESI- modes (*P* < 0.05, Figures 1(a) and 1(c)). These models were then assessed by permutation analysis, and all permuted *R*^2^s were below or around 0.6, and all permuted *Q*^2^s were below 0, which means that all *R*^2^s and *Q*^2^s are lower than the original on the right (Figures 1(b) and 1(d)). Thus, this suggests that these model fittings were valid and predictive. Finally, a total of 35 metabolites including 15 in the ESI+ mode and 20 in the ESI- mode responsible for distinguishing MAR patients from health controls were detected by UHPLC-MS analysis. The contribution plot ranks metabolites by their contribution to the model which is shown as a VIP. The top 10 metabolites with the highest VIP scores were identified as the most potential discriminant metabolites and 6 related metabolic pathways are listed in Table 2. According to metabolic pathway analyses, the most important pathways were arginine and proline metabolism, glycerophospholipid metabolism, sphingolipid metabolism, and fatty acid metabolism (Figure 2).

### 3.3. Metabolomics Profiling of MSAR vs. Health Controls

**Figure 3:**
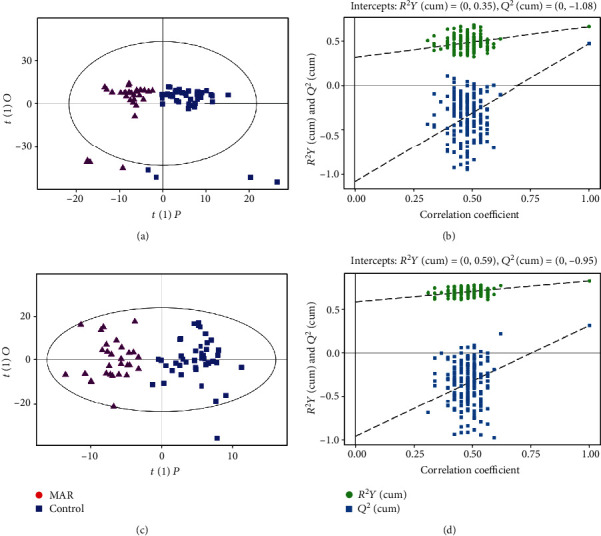
Metabolomics analysis of serum samples from the MSAR group and control group. Score plot with OPLS-DA in the ESI+ (a) and ESI- (c) modes in MSAR patients and controls. Permutation test of the OPLS-DA model in the ESI+ (b) and ESI- (d) modes. The values of *R*^2^*Y* and *Q*^2^ represent the goodness of fit and predictability of the model, respectively. OPLS-DA: orthogonal partial least square discriminant analysis; ESI: electrospray ionization; MSAR: moderate to severe allergic rhinitis.

**Figure 4:**
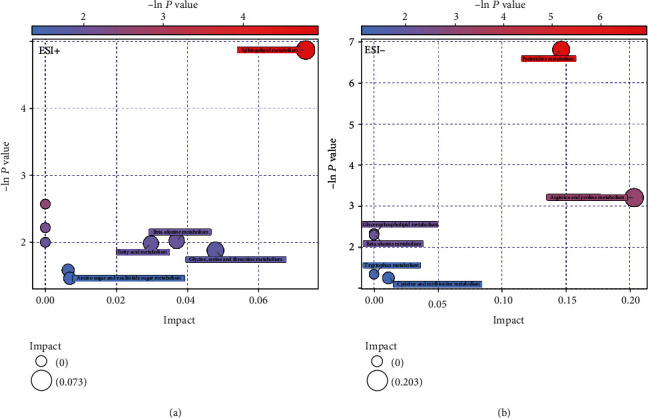
Metabolic function bubble chart based on the serum metabolomics profiles between MSAR patients and controls in the ESI+ (a) and ESI- (b) modes. ESI: electrospray ionization; MSAR: moderate to severe allergic rhinitis.

MSAR patients had different serum metabolic profiles in comparison with health controls by UHPLC-MS analysis in both the ESI+ and ESI- modes (*P* < 0.05, Figures 3(a) and 3(c)). The permutation analysis results showed that the model fittings were valid and predictive (Figures 3(b) and 3(d)). Compared to the control group, 59 metabolites including 29 in the ESI+ mode and 30 in the ESI- mode were expressed at significantly different levels in the MAR group. Results of the top 10 potential discriminant metabolites and 9 related metabolic pathways are listed in Table 3. The most important pathways including sphingolipid metabolism, pyrimidine metabolism, and arginine and proline metabolism are revealed in Figure 4.

### 3.4. Metabolomics Profiling of MSAR vs. MAR

**Figure 5:**
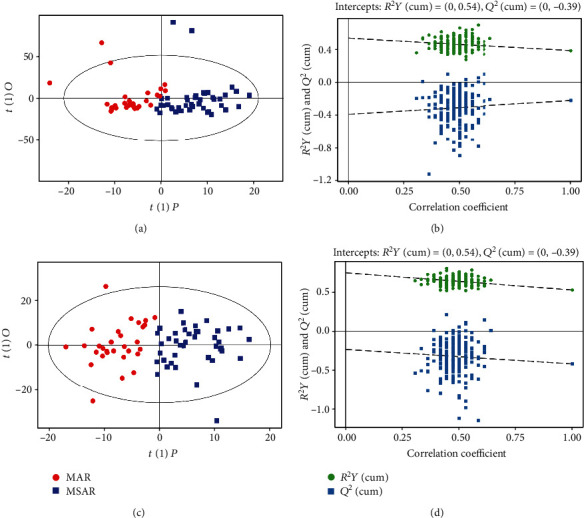
Metabolomics analysis of serum samples from the MSAR group and MAR group. Score plot with OPLS-DA in the ESI+ (a) and ESI- (c) modes in MSAR patients and MAR. Permutation test of the OPLS-DA model in the ESI+ (b) and ESI- (d) modes. The values of *R*^2^*Y* and *Q*^2^ represent the goodness of fit and predictability of the model, respectively. OPLS-DA: orthogonal partial least square discriminant analysis; ESI: electrospray ionization; MAR: mild allergic rhinitis; MSAR: moderate to severe allergic rhinitis.

**Figure 6:**
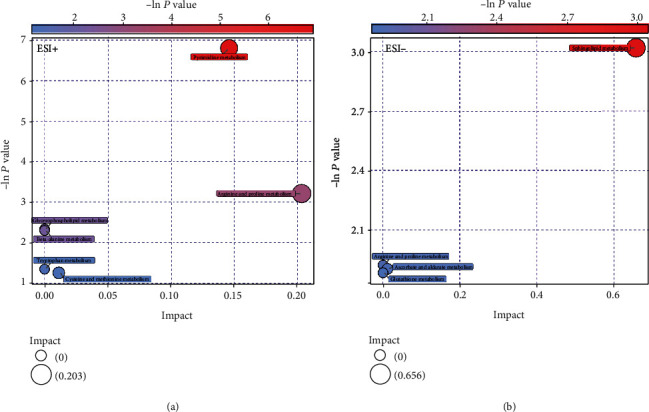
Metabolic function bubble chart based on the serum metabolomics profiles between MSAR patients and MAR in ESI+ (a) and ESI- (b) modes. ESI: electrospray ionization; MAR: mild allergic rhinitis; MSAR: moderate to severe allergic rhinitis.

In this study, AR patients were grouped into MSAR patients and MAR patients according to ARIA criteria, and the metabolic differences of these patients were further analyzed. As shown in Figure 5, the serum metabolomics profiles of MSAR patients and MAR patients were significant different form each other in both ion modes (*P* < 0.05, Figures 5(a) and 5(c)). The permutation analysis results exhibited good validation and predictability (Figures 5(b) and 5(d)). Compared with the MAR group, 30 metabolites including 17 in the ESI+ mode and 13 in the ESI- mode were detected at significantly different concentrations in the MSAR group. Results of the top 10 potential discriminant metabolites and 8 related metabolic pathways are displayed in Table 4. The most important pathways including fatty acid metabolism and sphingolipid metabolism are revealed in Figure 6.

### 3.5. Metabolomics Profiling and Severity of AR

**Figure 7:**
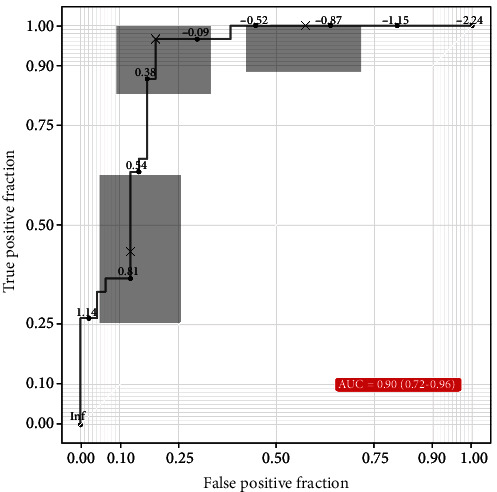
Composite ROC analysis of sarcosine, S1P, cytidine, and linoleic acid for predicting the disease severity in HDM-induced AR. AR: allergic rhinitis; S1P: sphingosine-1-phosphate; HDM: house dust mite; ROC: receiver operating characteristics.

The distinctive metabolites among the three groups with good predictability (AUC > 0.7) were included in Spearman's correlation analysis to evaluate their correlation with the severity of AR. As presented in Table 5, sarcosine, sphingosine-1-phosphate (S1P), and cytidine levels were positively correlated with TNSS and VAS in AR patients (*P* < 0.05). However, linoleic acid levels were negatively correlated with TNSS and VAS (*P* < 0.05). In order to evaluate the prediction power of four significantly distinctive metabolites in reflecting the disease severity, we performed a single-composite ROC analysis. The result showed that the composite predictor exhibited good accuracy and utility (AUC = 0.90, *P* < 0.001) (Figure 7).

## 4. Discussion

In the current prospective cohort study, we described a novel application of metabolomics in identifying the serum metabolic signatures and assessing the association between the distinctive metabolites and the severity of HDM-induced AR. The OPLS-DA model showed that obvious discriminators between patients with different disease severity and health controls. Thirty-five and 59 metabolites responsible for differentiating MAR and MSAR patients from health controls, respectively, were identified. In addition, 30 metabolites were found to be responsible for discriminating MSAR patients from MAR patients. After analyzing the relationships between the major discriminative metabolites and clinical parameters of patients, we observed that sarcosine, sphingosine-1-phosphate, cytidine, and linoleic acid levels were associated with the disease severity. These results showed that the identified potential serum metabolites might be useful for diagnosing HDM-induced AR and developing objective indicators for evaluating its severity. We will next discuss the most significant metabolites and related metabolic pathways, which may help us to better understand the underlying pathogenesis of HDM-induced AR and monitor its disease severity.

Most importantly, arginine and proline metabolism pathway was significantly perturbed among the most affected pathways in HDM-induced AR patients. Arginine and proline metabolism is of particular importance in the nitric oxide synthesis and integrally links to cellular respiration, metabolism, and inflammation [22, 23]. A recent publication detected arginine and proline metabolism significant perturbations in the serum of commuters following traffic pollution exposure, and the researchers considered that arginase and proline metabolism dysfunction were strongly associated with oxidative stress and inflammation in the air pollution toxicity [24]. Yang et al. [25] found that the levels of arginine and its downstream products, such as ornithine, citrulline, creatine, creatinine, hydroxyproline, and sarcosine, were higher in the serum of asthma patients than in health controls, and they held that arginine and proline metabolism was the most important pathway in the development of asthma. Consistent with the previous reports, we also observed that the levels of sarcosine and creatinine were higher in the serum of HDM-induced AR patients than health controls, and the levels of sarcosine correlated positively with TNSS and VAS. Arginine is an essential amino acid related to endothelial function, inflammation, and airway hyperresponsiveness, and higher levels of arginine and its downstream products can regulate T cell function and promote its activity, which act critical roles in several inflammatory diseases, including asthma and AR [22, 23, 26]. Therefore, we speculated that arginine and proline metabolism might be involved in the development of HDM-induced AR and sarcosine could roughly be related to the disease severity.

Our results also provide evidence that the sphingolipid metabolism alteration is involved in the occurrence and progression of HDM-induced AR. Sphingolipids are ubiquitous components of the cell membrane and play an important role in cell growth, inflammation, and tissue remodeling [27, 28]. Among the numerous sphingolipids, S1P has received the greatest attention in allergic diseases and autoimmune diseases, as it has been implicated in the modulation of a variety of cell responses such as immune cell proliferation, differentiation, and regulation [27, 29]. A previous study reported that S1P upregulated the cytokine production, such as IL-12, IL-23, and IL-27, in activated murine bone marrow-derived dendritic cells, and it might serve as a novel therapeutic target in the treatment of several inflammatory diseases [30]. In another study, researchers found that the plasma levels of SIP were elevated in cystic fibrosis patients, and S1P levels correlated with routine laboratory parameters, lung function, and clinical symptoms [31]. Kowal et al. [29] analyzed targeted metabolites in the serum of 22 allergic asthma patients and 11 allergic rhinitis patients and found that the sphingolipid metabolism was altered and the biosynthesis of S1P was augmented. In the present study, we observed that sphingolipid metabolism was disturbed and the S1P levels elevated in the HDM-induced AR patients, and the S1P levels were correlated positively with the disease severity, which was in accordance with the results in previous publications [29, 30]. Our results support the hypothesis that alterations in serum metabolites reflect the chronic activation of the immune system in AR patients and that the disease severity is consistent with greater activation of the immune system. However, the mechanism underlying these manipulations has not been well clarified.

We firstly found that cytidine, identified from UHPLC-MS analysis, was associated with HDM-induced AR, and it might be a novel marker and potential therapeutic target. In our study, we observed that the levels of cytidine were elevated in the MAR and MSAR groups, and the concentrations of cytidine were positively correlated with TNSS and VAS. Cytidine, a pyrimidine molecule, is considered the precursor of the cytidine triphosphate (CTP), which is vital in the synthesis, interconversion, and degradation of DNA, RNA, and lipids [32, 33]. Previous studies have found that abnormalities of pyrimidine metabolism could influence cell growth, development, and differentiation of T cell and B cell [34]. A recent report demonstrated that interference of pyrimidine metabolism affected murine lymphocyte proliferation in vitro and attenuated the severity of experimental autoimmune arthritis [35]. Another study observed that the concentrations of 5,6-dihydorthymine were higher in the serum of current asthma patients compared with health controls, and the researchers believed that the alteration of pyrimidine metabolism might have relevance for asthma pathophysiology [36]. These events suggested that pyrimidine metabolism might play a pivotal role in autoimmune diseases and allergic diseases. Therefore, we ultimately believed that cytidine was associated with AR and that it might serve as a promising metabolic biomarker for assessing its disease severity.

Interestingly, we also found that the fatty acid metabolism was dysregulated in all OPLS-DA models. In recent years, growing evidence suggested that the fatty acid metabolism played important roles in the modulation of immune responses [37]. Most researchers hold that unsaturation fatty acids, especially polyunsaturated fatty acids, exhibited potential protective effects on allergic inflammation, while saturation fatty acids promoted the inflammatory response [38]. In a recent animal experimental study, Lee et al. [39] observed that oleic acid had antiasthmatic effects, including downregulation of inflammatory cells and eosinophils in bronchial alveolar lavage fluid, IgE in serum. Several in vitro studies also demonstrated that unsaturation fatty acids could exert immunosuppressive effects on T cells, such as reducing its proliferation and activation in a dose-dependent manner [37, 40]. However, saturation fatty acids, such as palmitic acid, have been described as essential factors promoting T cell activation and cytokine secretion [41]. In addition, considerable evidence shown that polyunsaturated fatty acids could modify mast cell function and suppress its activation and then reduce the production of cytokine or chemokine receptors [42]. Therefore, we suppose that fatty acid metabolism may be essential in the development of AR. In the current study, the concentrations of several unsaturated fatty acids (linoleic acid, arachidic acid, and *trans*-vaccenic acid) were lower in the serum of MAR or MSAR patients in comparison with health controls, while the concentrations of palmitic acid were elevated. Moreover, the levels of linoleic acid were correlated negatively with TNSS and VAS. Our results were in line with most previous studies. However, further studies should be conducted to confirm these results and to clarify the underlying mechanism of AR subtypes.

We acknowledge several limitations in our study which may affect the clinical applications of obtained results. First, the total sample sizes were relatively small and a validation cohort study was needed to confirm the conclusions. Second, the recruited participants were from a single center with the same ethnicity and region, which might limit the applicability of our findings. Third, only one biological sample (serum) was used in the present study; future studies should collect other biological samples, such as urine and nasal lavage fluid, to further verify whether the identified differential metabolites were associated with AR. Last, we did not compare serum metabolites between moderate AR and severe AR patients, but it does not mean that there are no differential metabolites. Future multicenter prospective clinical studies with larger sample sizes utilizing untargeted and targeted metabolomics will be important to support and extend our present findings.

## 5. Conclusion

Our results suggest that serum metabolomics approaches can be successfully used to discriminate MSAR patients, from MAR patients and health controls, and establish a metabolite signature associated with the severity of HDM-induced AR. These results will be useful for diagnosing HDM-induced AR and developing objective indicators for evaluating the disease severity.

## Figures and Tables

**Table 1:** Clinical characteristics of participants.

Variable	Control (*n* = 29)	MAR (*n* = 30)	MSAR (*n* = 42)	*P* value
Gender (male/female)	14/15	17/13	22/20	0.914
Age (years)	28.5 ± 8.5	28.2 ± 9.6	30.4 ± 8.4	0.518
BMI (kg/m^2^)	22.2 ± 1.8	22.8 ± 1.8	22.1 ± 1.6	0.224
Disease duration (years)	NA	5.5 ± 2.8	4.9 ± 2.2	0.300
Total IgE (IU/mL)	79.2 ± 29.1	212.4 ± 83.6	430.4 ± 155.7	<0.001
Specific IgE to HDM (IU/mL)	0.2 ± 0.1	0.7 ± 0.2	5.8 ± 3.9	<0.001
Blood eosinophil counts (cells/*μ*L)	81.2 ± 24.0	176.1 ± 97.8	451.2 ± 192.4	<0.001
TNSS	1.2 ± 0.7	3.9 ± 1.0	9.5 ± 1.7	<0.001
VAS	1.3 ± 0.6	3.1 ± 0.9	6.9 ± 1.5	<0.001

MAR: mild allergic rhinitis; MSAR: moderate-severe allergic rhinitis; BMI: body mass index; HDM: house dust mite; TNSS: total nasal symptom score; NA: not applicable.

**Table 2:** Top ten metabolites with different variations discriminating MAR patients from health controls.

Metabolites	Ion mode	VIP	*P* value	Regulation	AUC	Pathway
Sarcosine	Positive	3.24	0.005	Up	0.77	Arginine and proline metabolism
Palmitic acid	Positive	2.74	0.000	Up	0.87	Fatty acid metabolism
5′-Methylthioadenosine	Positive	2.58	0.002	Up	0.60	Cysteine and methionine metabolism
Triethanolamine	Negative	2.16	0.047	Up	0.79	Glycerophospholipid metabolism
5-Methoxyindoleacetate	Negative	1.86	0.004	Up	0.75	Tryptophan metabolism
*trans*-Vaccenic acid	Positive	1.79	0.004	Down	0.93	Fatty acid metabolism
Creatinine	Negative	1.73	0.000	Up	0.78	Arginine and proline metabolism
S1P	Negative	1.70	0.019	Up	0.91	Sphingolipid metabolism
Arachidic acid	Positive	1.62	0.014	Down	0.54	Fatty acid metabolism
L-Methionine	Negative	1.61	0.022	Down	0.49	Cysteine and methionine metabolism

MAR: mild allergic rhinitis; VIP: variable importance for project; AUC: area under the curve; S1P: sphingosine-1-phosphate.

**Table 3:** Top ten metabolites with different variations discriminating MSAR patients from health controls.

Metabolites	Ion mode	VIP	*P* value	Regulation	AUC	Pathway
S1P	Positive	1.97	0.005	Up	0.89	Sphingolipid metabolism
2-Oxoadipic acid	Negative	2.36	0.002	Up	0.68	Tryptophan metabolism
Phosphorylcholine	Negative	2.19	0.002	Up	0.86	Glycerophospholipid metabolism
Cytidine	Negative	2.01	0.000	Up	0.83	Pyrimidine metabolism
Betaine	Positive	1.97	0.000	Up	0.84	Glycine, serine and threonine metabolism
Sarcosine	Negative	1.95	0.002	Up	0.92	Arginine and proline metabolism
1,3-Diaminopropane	Positive	1.85	0.007	Up	0.61	Beta-alanine metabolism
Taurocholic acid	Positive	1.82	0.006	Down	0.74	Taurine and hypotaurine metabolism
Linoleic acid	Negative	1.78	0.026	Down	0.79	Fatty acid metabolism
*cis*-9-Palmitoleic acid	Positive	1.75	0.005	Up	0.64	Fatty acid metabolism

MSAR: moderate-severe allergic rhinitis; VIP: variable importance for project; AUC: area under the curve; S1P: sphingosine-1-phosphate.

**Table 4:** Top ten metabolites with different variations discriminating MSAR from MAR.

Metabolites	Ion mode	VIP	*P* value	Regulation	AUC	Pathway
Linoleic acid	Positive	2.79	0.049	Down	0.77	Fatty acid metabolism
Betaine	Positive	2.24	0.034	Up	0.60	Glycine, serine, and threonine metabolism
Coumarin	Positive	2.11	0.012	Down	0.63	Phenylpropanoid biosynthesis
S1P	Negative	2.06	0.009	Up	0.72	Sphingolipid metabolism
Palmitoleic acid	Positive	1.97	0.016	Down	0.75	Fatty acid metabolism
*trans*-Vaccenic acid	Positive	1.89	0.003	Down	0.65	Fatty acid metabolism
D-Glucurono-6,3-lactone	Negative	1.88	0.000	Up	0.59	Ascorbate and aldarate metabolism
Sarcosine	Negative	1.83	0.007	Up	0.91	Arginine and proline metabolism
Cytidine	Positive	1.79	0.017	Up	0.74	Pyrimidine metabolism
Pyroglutamic acid	Negative	1.72	0.014	Down	0.81	Glutathione metabolism

MAR: mild allergic rhinitis; MSAR: moderate-severe allergic rhinitis; VIP: variable importance for project; AUC: area under the curve; S1P: sphingosine-1-phosphate.

**Table 5:** Correlation of serum metabolites with severity of HDM-induced AR.

Metabolites	TNSS		VAS	
	*r*	*P* value	*r*	*P* value
Sarcosine	0.551	0.012	0.376	0.040
Palmitic acid	0.489	0.137	0.413	0.107
Triethanolamine	-0.212	0.170	0.301	0.019
Betaine	0.431	0.049	0.298	0.202
5-Methoxyindoleacetate	-0.204	0.765	0.376	0.046
*trans*-Vaccenic acid	-0.312	0.031	-0.178	0.099
Creatinine	0.702	0.129	0.561	0.049
S1P	0.821	0.004	0.673	0.030
Phosphorylcholine	0.378	0.418	0.277	0.031
Cytidine	0.598	0.028	0.312	0.017
Diethanolamine	0.242	0.782	0.134	0.458
*cis*-9,10-Epoxystearic acid	0.366	0.232	-0.221	0.022
Taurocholic acid	-0.207	0.651	-0.319	0.562
Linoleic acid	-0.792	0.031	-0.493	0.041
Palmitoleic acid	-0.377	0.052	-0.274	0.093
Pyroglutamic acid	-0.134	0.202	-0.307	0.089

AR: allergic rhinitis; TNSS: total nasal symptom score; VAS: visual analogue scale; HDM: house dust mite; S1P: sphingosine-1-phosphate.

## Data Availability

The data used to support the findings of this study are available from the corresponding author upon request.

## References

[1] Ai J., Xie Z., Qing X. (2018). Clinical effect of endoscopic vidian neurectomy on bronchial asthma outcomes in patients with coexisting refractory allergic rhinitis and asthma. *American Journal of Rhinology & Allergy*.

[2] Chen X., Xie Z. H., Lv Y. X. (2016). A proteomics analysis reveals that A2M might be regulated by STAT3 in persistent allergic rhinitis. *Clinical and Experimental Allergy*.

[3] Meng Y., Wang C., Zhang L. (2019). Recent developments and highlights in allergic rhinitis. *Allergy*.

[4] Shen Z., Tan G., Zhong Z., Ding S., Wang F. (2019). Interactive network platform improves compliance and efficacy of subcutaneous immunotherapy for patients with allergic rhinitis. *Patient Preference and Adherence*.

[5] Zhang Y., Zhang L. (2019). Increasing prevalence of allergic rhinitis in China. *Allergy, Asthma & Immunology Research*.

[6] Brożek J. L., Bousquet J., Agache I. (2017). Allergic Rhinitis and its Impact on Asthma (ARIA) guidelines--2016 revision. *The Journal of Allergy and Clinical Immunology*.

[7] Del Cuvillo A., Santos V., Montoro J. (2017). Allergic rhinitis severity can be assessed using a visual analogue scale in mild, moderate and severe. *Rhinology*.

[8] Hou J., Lou H., Wang Y. (2018). Nasal ventilation is an important factor in evaluating the diagnostic value of nasal nitric oxide in allergic rhinitis. *International Forum of Allergy & Rhinology*.

[9] Han M. W., Kim S. H., Oh I., Kim Y. H., Lee J. (2019). Serum IL-1*β* can be a biomarker in children with severe persistent allergic rhinitis. *Allergy, Asthma and Clinical Immunology*.

[10] Pirayesh A., Shahsavan S., Zargari Samani O. (2019). Differential expression of Fas in moderate/severe and mild persistent allergic rhinitis and its correlation with pathological parameters. *American Journal of Rhinology & Allergy*.

[11] Chen C., Luo F., Wu P. (2020). Metabolomics reveals metabolite changes of patients with pulmonary arterial hypertension in China. *Journal of Cellular and Molecular Medicine*.

[12] Zhan X., Long Y., Lu M. (2018). Exploration of variations in proteome and metabolome for predictive diagnostics and personalized treatment algorithms: innovative approach and examples for potential clinical application. *Journal of Proteomics*.

[13] Adamko D. J., Nair P., Mayers I., Tsuyuki R. T., Regush S., Rowe B. H. (2015). Metabolomic profiling of asthma and chronic obstructive pulmonary disease: a pilot study differentiating diseases. *The Journal of Allergy and Clinical Immunology*.

[14] Ning P., Zheng Y., Luo Q. (2018). Metabolic profiles in community-acquired pneumonia: developing assessment tools for disease severity. *Critical Care*.

[15] Spertini F. (2020). Metabolomics and allergy: opening Pandora's box. *The Journal of Allergy and Clinical Immunology*.

[16] Dunn W. B., Broadhurst D., Begley P. (2011). Procedures for large-scale metabolic profiling of serum and plasma using gas chromatography and liquid chromatography coupled to mass spectrometry. *Nature Protocols*.

[17] Liu S., Liang Y.-Z., Liu H.-T. (2016). Chemometrics applied to quality control and metabolomics for traditional Chinese medicines. *Journal of Chromatography. B, Analytical Technologies in the Biomedical and Life Sciences*.

[18] Zhao H., Cheng N., Wang Q. (2019). Effects of honey-extracted polyphenols on serum antioxidant capacity and metabolic phenotype in rats. *Food & Function*.

[19] Kuhl C., Tautenhahn R., Böttcher C., Larson T. R., Neumann S. (2012). CAMERA: an integrated strategy for compound spectra extraction and annotation of liquid chromatography/mass spectrometry data sets. *Analytical Chemistry*.

[20] Yang Y., Wu Z., Li S. (2020). Targeted blood metabolomic study on retinopathy of prematurity. *Investigative Ophthalmology & Visual Science*.

[21] Wang W., Zhao L., He Z. (2018). Metabolomics-based evidence of the hypoglycemic effect of Ge-Gen-Jiao-Tai-Wan in type 2 diabetic rats via UHPLC-QTOF/MS analysis. *Journal of Ethnopharmacology*.

[22] King N. E., Rothenberg M. E., Zimmermann N. (2004). Arginine in asthma and lung inflammation. *The Journal of Nutrition*.

[23] Scott J. A., Grasemann H. (2014). Arginine metabolism in asthma. *Immunology and Allergy Clinics of North America*.

[24] Liang D., Ladva C. N., Golan R. (2019). Perturbations of the arginine metabolome following exposures to traffic-related air pollution in a panel of commuters with and without asthma. *Environment International*.

[25] Quan-Jun Y., Jian-Ping Z., Jian-Hua Z. (2017). Distinct metabolic profile of inhaled budesonide and salbutamol in asthmatic children during acute exacerbation. *Basic & Clinical Pharmacology & Toxicology*.

[26] Xu W., Comhair S. A. A., Janocha A. J. (2017). Arginine metabolic endotypes related to asthma severity. *PLoS One*.

[27] Mohammed S., Harikumar K. B. (2017). Sphingosine 1-phosphate: a novel target for lung disorders. *Frontiers in Immunology*.

[28] Rivera J., Proia R. L., Olivera A. (2008). The alliance of sphingosine-1-phosphate and its receptors in immunity. *Nature Reviews. Immunology*.

[29] Kowal K., Żebrowska E., Chabowski A. (2019). Altered sphingolipid metabolism is associated with asthma phenotype in house dust mite-allergic patients. *Allergy, Asthma & Immunology Research*.

[30] Schaper K., Kietzmann M., Bäumer W. (2014). Sphingosine-1-phosphate differently regulates the cytokine production of IL-12, IL-23 and IL-27 in activated murine bone marrow derived dendritic cells. *Molecular Immunology*.

[31] Halilbasic E., Fuerst E., Heiden D. (2020). Plasma levels of the bioactive sphingolipid metabolite S1P in adult cystic fibrosis patients: potential target for immunonutrition?. *Nutrients*.

[32] Chen H. W., Zhou W., Liao Y., Hu S. C., Chen T. L., Song Z. C. (2018). Analysis of metabolic profiles of generalized aggressive periodontitis. *Journal of Periodontal Research*.

[33] Zhu X.-R., Yang F.-Y., Lu J. (2019). Plasma metabolomic profiling of proliferative diabetic retinopathy. *Nutrition & Metabolism (London)*.

[34] Garavito M. F., Narváez-Ortiz H. Y., Zimmermann B. H. (2015). Pyrimidine metabolism: dynamic and versatile pathways in pathogens and cellular development. *Journal of Genetics and Genomics*.

[35] Peres R. S., Santos G. B., Cecilio N. T. (2017). Lapachol, a compound targeting pyrimidine metabolism, ameliorates experimental autoimmune arthritis. *Arthritis Research & Therapy*.

[36] Kelly R. S., Sordillo J. E., Lasky-Su J. (2018). Plasma metabolite profiles in children with current asthma. *Clinical and Experimental Allergy*.

[37] Wang X., Kulka M. (2015). N-3 polyunsaturated fatty acids and mast cell activation. *Journal of Leukocyte Biology*.

[38] Venter C., Meyer R. W., Nwaru B. I. (2019). EAACI position paper: influence of dietary fatty acids on asthma, food allergy, and atopic dermatitis. *Allergy*.

[39] Lee S.-Y., Bae C.-S., Seo N.-S. (2019). Camellia japonica oil suppressed asthma occurrence via GATA-3 & IL-4 pathway and its effective and major component is oleic acid. *Phytomedicine*.

[40] Arita M. (2016). Eosinophil polyunsaturated fatty acid metabolism and its potential control of inflammation and allergy. *Allergology International*.

[41] Radzikowska U., Rinaldi A. O., Çelebi Sözener Z. (2019). The influence of dietary fatty acids on immune responses. *Nutrients*.

[42] Yu G., Björkstén B. (1998). Polyunsaturated fatty acids in school children in relation to allergy and serum IgE levels. *Pediatric Allergy and Immunology*.

